# 肺部巨大球形占位——颅内血管外膜细胞瘤肺转移1例

**DOI:** 10.3779/j.issn.1009-3419.2012.09.09

**Published:** 2012-09-20

**Authors:** 雪峰 孙, 巍 钟

**Affiliations:** 100730 北京，北京协和医院呼吸内科 Department of Respiratory Medicine, Peking Union Medical College Hospital, Beijing 100730, China

## 1 临床资料

患者，女，61岁，因咯血3月，咳嗽、喘憋2周于2011年1月7日收住我院。患者于3个月前无明显诱因咯少量暗红色血痰，自行好转，未诊治。2周前受凉后出现干咳，无发热、盗汗或咯血，咳嗽症状进行性加重，并逐渐出现喘憋，活动后明显。患者既往曾于2年前因右中颅窝、颞下窝占位行手术切除，术后病理诊断为血管外膜细胞瘤，术后行放疗，当时胸片未见异常，术后规律随诊头颅磁共振未发现异常。入院查体：体温36.8 ℃，脉搏87次/分，呼吸19次/分，血压110/80 mmHg（1 mmHg=0.133 kPa）。浅表淋巴结无肿大，右肺底呼吸音减弱，叩浊，双肺未闻及干湿啰音，心、腹查体无异常。实验室检查：血常规：白细胞8.54×10^9^/L，中性粒细胞73.3%，血红蛋白111 g/L，血小板382×10^9^/L；血沉102 mm/hr，结核抗体弱阳性，G试验63 pg/mL。肿瘤标志物（包括癌胚抗原、CA199、CA242、组织多肽抗原、角质蛋白19片段和神经元烯醇化酶）均为阴性。痰液检查：细菌、真菌、抗酸菌涂片和培养均为阴性。胸片示右胸部巨大团块影，右侧肋隔角浅钝；胸腹增强CT示右肺下叶球形软组织密度肿块影，边缘光滑，直径约6.0 cm，右中间段及右下叶支气管阻塞，双肺尚可见多发结节影，纵隔淋巴结略肿大，右侧胸膜增厚；肝内多发低密度灶（图 1）。头颅增强核磁检查与既往比较无变化。PET/CT示肺部肿物低葡萄糖代谢，多发结节和淋巴结正常代谢，肝内病灶正常代谢。行支气管镜检查：右中间段支气管内新生物，表面红色，血供丰富，阻塞管腔（图 2），镜下肿物活检病理示纤维素样坏死物。行CT引导下经皮肺穿，病理检查示炎性肉芽组织伴坏死组织。再次行支气管镜下活检，活检病理为见梭形细胞聚集，较多核分裂相，符合梭形细胞瘤，结合病史符合脑血管外膜细胞瘤肺转移（图 3）。给予异环磷酰胺2 g第1天-5天静脉滴注、表柔比星50 mg第1天、2天静脉滴注方案化疗，每3周为1个周期，共行6程化疗。6个疗程后评估病情稳定，但化疗结束2个月后评估病情再次进展。

**1:**
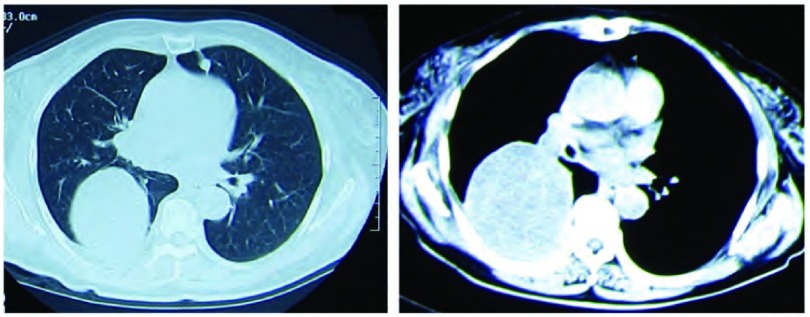
胸部增强CT示右下叶直径约6 cm的球形占位，边界光滑无分叶，呈不均匀增强。此层面CT上尚可见左舌叶小结节影。 Contrast-enhanced chest CT scan shows a spherical mass 6cm in diameter in right lower lobe. It has a smooth margin without lobulation, and is irregularly enhanced. A small nodule is also seen in left lingular lobe.

**2:**
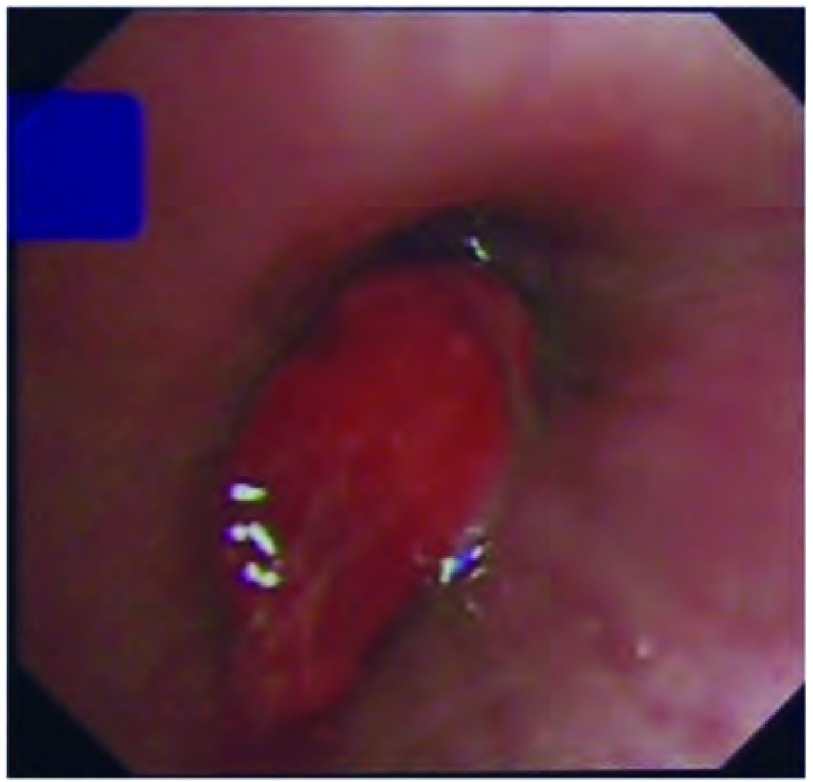
纤维支气管镜下见右中间段支气管内新生物，表面红色，血供丰富，阻塞管腔。 Fibrobronchoscopy reveals a blood-red neoplasm in right intermediate bronchus, which has rich blood supply and blocks the lumen.

**3:**
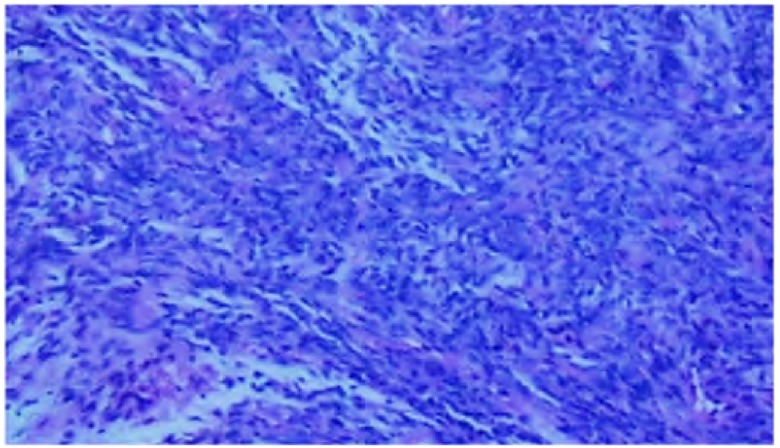
病理检查见梭形细胞聚集，较多核分裂相，符合梭形细胞瘤（HE, ×100）。 Pathological exam: spindle cells are seen aggregated, and have many mitoses, conforming to spindle cell tumor (HE, ×100).

## 2 讨论

此患者临床表现为咳嗽、咯血、气短，CT提示右下叶巨大圆球形占位，且不均匀增强，此外双肺尚有多发小结节，因此一度将肺癌列为首要考虑。但从占位形态来看，边界非常光滑，未见分叶、毛刺、空洞等肺癌常见影像表现，肿瘤标志物均正常，不太支持肺癌诊断。肺淋巴瘤或淋巴增殖性疾病可表现为球形结节团块，一般边界不规则，偶可见支气管充气征。正因为其球形占位边界光滑，提示有包膜包被，这更符合肺部良性肿瘤，如腺瘤、平滑肌瘤、纤维瘤等，但良性肿瘤一般不会发生转移。感染性疾病中球形肺炎可呈球形，但一般边缘毛糙，且往往可见支气管充气征，临床上一般有发热、咳痰、白细胞增高等表现；慢性感染性疾病可表现为球形占位的主要是结核球和真菌球，两者均由纤维组织包绕，呈非侵袭状态，多由之前活动性感染发展而来。结缔组织疾病中，可表现为肺部球形占位的以类风湿结节最为多见，边界光滑，一般直径 < 5 cm，多数表现为多发结节。其它如动静脉畸形，也有可能呈球形，增强CT可助鉴别。此患者既往有脑血管外膜细胞瘤病史，虽然前两次活检均未能明确诊断，但最终经活检证实肺内球形占位同样为血管外膜细胞瘤。由于未能获得既往颅内肿瘤的病理标本，因此不能比较两个部位肿瘤的同源性，为本研究的不足之处。但根据肿瘤发生的先后顺序，以及此次同时发现肝内转移灶，考虑颅内血管外膜细胞瘤发生肝、肺转移是更为合理的解释。

血管外膜细胞瘤是一种罕见的软组织血管肿瘤，以侵犯骨髂、肌肉和皮肤为主，占所有神经系统肿瘤的0.4%-1%^[1]^。血管外膜细胞瘤生长缓慢，通常存在数年后方确诊，临床往往表现为局部压迫症状。尽管如此，血管外膜细胞瘤在生物特性上仍具有很强的侵袭性，即使大体切除仍有可能局部复发，或出现中枢神经系统之外的转移。Mena等^[2]^对94例中枢神经系统血管外膜细胞瘤患者的回顾性研究表明，其转移率高达27%，而骨、肺和肝是最易出现转移的部位。颅外转移平均发生在初始治疗后8年，最长的甚至有间隔22年的报道^[3]^，因此对于血管外膜细胞瘤的患者应长期随诊。

此病例头颅核磁未发现原发肿瘤复发，而PET检查结果为阴性，这也给判断肺占位良恶性造成一定困难。尽管大多数相关文献均报道血管外膜细胞瘤的PET摄取值增高，但也有摄取值不增高的报道，提示对于血管外膜细胞瘤，无法用PET来鉴别肿瘤的良恶性^[4, 5]^。

手术是当前治疗血管外膜细胞瘤最有效的措施，术后放疗有助于降低复发率并延长生存时间。对于无法完全切除的原发肿瘤，姑息性手术加术后放疗是推荐的治疗方法。对于已经发生远处转移的肿瘤，通常使用以阿霉素为基础的化疗方案进行化疗，但疗效差强人意^[6, 7]^。Park等^[8]^选用阿霉素联合异环磷酰胺、异环磷酰胺单药或吉西他滨联合多西他赛用以治疗晚期血管外膜细胞瘤，这5例患者的平均无进展生存期仅为6.1个月（1.6个月-9个月）；本文此例患者的治疗反应与此类似。作为对比，Park等^[8]^最近还尝试了替莫唑胺联合贝伐单抗治疗晚期血管外膜细胞瘤，14例患者平均无进展生存时间延长至9.7个月，优于传统化疗方案。此治疗方案的优越性尚需更大规模的前瞻性研究来证实。
